# Cloud-Based AutoML Classification of Malignant and Non-malignant Colorectal Findings on Abdominal Computed Tomography: A Proof-of-Concept Study

**DOI:** 10.7759/cureus.116802

**Published:** 2026-09-23

**Authors:** Mark A Bachir, Neel Nawathey, Alexander S Bachir, Akshay J Reddy, Jacob Webster, Simran Gill, Rakesh Patel

**Affiliations:** 1 Internal Medicine, California Northstate University College of Medicine, Elk Grove, USA; 2 Osteopathic Medicine, Touro University California, Vallejo, USA; 3 Internal Medicine, California Health Sciences University, Fresno, USA; 4 Medicine, California University of Science and Medicine, Colton, USA; 5 Medicine, The University of Oklahoma, Norman, USA; 6 Medicine, University of California, Los Angeles, Los Angeles, USA; 7 Internal Medicine, East Tennessee State University Quillen College of Medicine, Johnson City, USA

**Keywords:** artificial intelligence, automl, colorectal cancer, computed tomography, deep learning, image classification, machine learning, medical imaging

## Abstract

Colorectal cancer (CRC) is a major cause of cancer-related morbidity and mortality worldwide, and computed tomography (CT) plays an important role in its clinical evaluation and staging. However, CT interpretation may be influenced by lesion conspicuity, acquisition characteristics, and reader experience. Artificial intelligence (AI)-based image analysis may provide a reproducible adjunct to conventional radiologic assessment. This study evaluated a cloud-based automated machine-learning image-classification model for distinguishing malignant from non-malignant abdominal CT images. Images were obtained from the publicly available ColonCancerCT-2025 dataset, which contains CT imaging and clinical information from 40 patients, including 12 malignant and 28 non-malignant cases. For the present analysis, 3,827 CT images were included. The images were divided into training, validation, and test sets in an approximately 80:10:10 ratio, comprising 3,062 training images, 383 validation images, and 382 test images. Patient-level separation among these partitions could not be confirmed from the retained study workflow. A single-label binary image-classification model was developed using Google Cloud AutoML (Google LLC, Mountain View, CA, USA). Model performance was evaluated using average precision, precision, recall, precision-recall analysis, confidence-threshold behavior, and a normalized confusion matrix. A confidence threshold of 0.50 was used for the primary performance assessment. At this threshold, the classifier achieved an overall average precision of 0.998, with precision and recall of approximately 99%. Class-specific average precision was 0.998 for malignant images and 0.999 for non-malignant images. The normalized confusion matrix demonstrated correct classification of 97% of malignant images and 100% of non-malignant images. Representative correctly classified images received prediction probabilities of 0.947 for the malignant class and 0.960 for the non-malignant class. These findings demonstrate the feasibility of cloud-based automated image classification for colorectal CT analysis; however, the limited number of patients, lack of confirmed patient-level separation between dataset partitions, absence of independent external testing, and image-level rather than examination-level evaluation substantially limit conclusions regarding clinical generalizability. Larger patient-level, multi-institutional studies are required before such a model can be considered for clinical decision support.

## Introduction

Colorectal cancer (CRC) remains a major global health burden. GLOBOCAN 2024 estimates indicate that approximately 2.04 million new CRC cases and 918,000 CRC deaths occurred worldwide in 2024, making CRC the third most commonly diagnosed malignancy and the second leading cause of cancer-related death globally [1]. The development of CRC reflects interactions among age, hereditary susceptibility, environmental exposures, and modifiable factors including obesity, physical inactivity, smoking, alcohol consumption, and dietary patterns [2]. Population-based screening can identify premalignant lesions and cancers at earlier stages and reduce CRC-associated morbidity and mortality [3]. Once cancer is identified, treatment selection depends on multiple factors, including tumor location, local extent, nodal involvement, metastatic disease, pathological characteristics, molecular features, and individual patient characteristics [4].

Imaging is central to the diagnosis, staging, treatment planning, and surveillance of patients with colorectal malignancy. Colonoscopy remains fundamental for direct visualization of the colorectal mucosa and enables tissue acquisition for histopathologic confirmation, while cross-sectional imaging provides complementary information regarding local and distant disease [4,5]. Computed tomography (CT) is routinely incorporated into colon cancer staging because of its widespread availability, rapid image acquisition, and ability to evaluate the primary tumor, adjacent structures, lymph nodes, and distant metastatic sites [4-6]. Magnetic resonance imaging and positron emission tomography/CT may provide additional targeted information in selected clinical settings [5].

Despite its clinical value, conventional CT has limitations in the characterization and staging of colorectal malignancy. Lesion conspicuity can vary according to tumor size, tumor location, bowel distention, contrast enhancement, reconstruction technique, surrounding anatomy, and tumor morphology. CT performance is also not uniform across staging tasks. Earlier systematic evaluation demonstrated substantially greater accuracy for detecting distant metastatic disease than for precise local tumor and nodal staging [6]. Nerad et al. subsequently reported high sensitivity for identifying tumor extension beyond the bowel wall but more modest specificity and limited diagnostic accuracy for nodal involvement [7]. More recent systematic reviews continue to demonstrate imperfect CT performance for high-risk features, including deep extramural invasion, extramural venous invasion, and nodal disease [8,9]. These limitations provide a rationale for investigating computational approaches that may extract reproducible imaging features that are difficult to consistently identify through visual interpretation alone.

Artificial intelligence (AI) has increasingly been investigated for quantitative analysis of medical images. Machine-learning algorithms can evaluate complex combinations of imaging characteristics associated with disease, while deep-learning systems can derive hierarchical representations directly from image data. Radiomics provides a complementary approach by transforming images into quantitative descriptors of shape, intensity, texture, and spatial heterogeneity. Within CRC imaging, AI and radiomics have been investigated for tumor detection, staging, prediction of metastatic risk, assessment of treatment response, and prognostic modeling [10]. The ability to analyze large quantities of image data reproducibly may allow AI systems to complement, rather than replace, conventional radiologic interpretation.

Recent investigations have demonstrated the potential of deep learning for direct CRC detection on routine CT examinations. Yao et al. developed a deep-learning system for CRC detection on contrast-enhanced CT performed without bowel preparation. Across internal, external, and real-world test cohorts, their system achieved receiver-operating-characteristic area under the curve (ROC-AUC) values ranging from 0.957 to 0.994 and improved radiologist accuracy in reader experiments [11]. Chen et al. subsequently evaluated a deep-learning system for CRC detection on routine non-contrast CT in multicenter and international cohorts. Their model achieved area-under-the-curve values of 0.967 to 0.996 across multicenter testing and improved radiologist sensitivity and specificity with AI assistance [12]. These studies demonstrate that clinically meaningful image patterns associated with colorectal malignancy can be identified even on CT examinations not specifically designed for CRC screening.

AI has also been studied for tasks extending beyond primary tumor detection. Bedrikovetski et al. evaluated a ResNet-50-based deep-learning model for prediction of lymph-node status from preoperative staging CT. Although the approach demonstrated the feasibility of automated nodal assessment, performance in independent testing illustrated the difficulty of translating promising model-development results to unseen patients [13]. CT-derived radiomic signatures have also been investigated for postoperative prognosis and survival prediction [14]. Li et al. developed a multisize convolutional neural network using preoperative CT features to predict recurrence and disease-free survival [15], while Hong et al. developed a hybrid machine-learning CT radiomics nomogram for prediction of cancer-specific survival after curative CRC resection [16]. More recently, machine-learning radiomics has been evaluated for prediction of primary tumor and nodal stages, further demonstrating that quantitative CT characteristics may contain information related to invasive tumor behavior [17].

Despite these advances, performance estimates for medical-imaging AI systems depend heavily on study design and data composition. Scanner hardware, reconstruction algorithms, contrast protocols, image resolution, labeling methods, disease prevalence, and patient characteristics may all influence model performance. Small or homogeneous datasets are particularly vulnerable to overly optimistic estimates when development and testing data are not completely independent. This issue is especially important for CT datasets containing many images from relatively few patients because adjacent images from a single CT examination may contain highly correlated anatomical information. Contemporary reporting guidance, including the Checklist for Artificial Intelligence in Medical Imaging (CLAIM), emphasizes transparent reporting of data sources, reference standards, partitioning methods, model evaluation, and external testing [18]. The TRIPOD+AI statement similarly emphasizes clear reporting of prediction-model development and evaluation to facilitate assessment of reproducibility, bias, and clinical applicability [19].

The objective of the present proof-of-concept study was to evaluate whether a cloud-based automated image-classification platform could distinguish malignant from non-malignant abdominal CT images obtained from the publicly available ColonCancerCT-2025 dataset [20]. Model behavior was assessed using average precision, precision, recall, precision-recall analysis, confidence-threshold analysis, a normalized confusion matrix, and representative image-level predictions. The study was intended to evaluate technical feasibility rather than establish independent clinical diagnostic performance.

## Materials and methods

Study design and data source

This retrospective computational proof-of-concept study evaluated a binary AI image-classification model using de-identified abdominal CT images obtained from the publicly available ColonCancerCT-2025 dataset [20]. The source dataset contains CT imaging and associated clinical information from 40 patients, including 12 patients categorized as malignant and 28 categorized as non-malignant. Original DICOM (Digital Imaging and Communications in Medicine) studies and processed image files are made available through the source dataset for computational research [20].

A total of 3,827 CT images were incorporated into the present model-development workflow. Each image was assigned one of two diagnostic labels provided by the source dataset: malignant or non-malignant. These dataset-supplied labels served as the reference standard for model development and evaluation. No new clinical adjudication, histopathologic review, or relabeling was performed by the investigators.

Images were eligible for the present analysis if they were included in the investigators' 3,827-image analysis set and carried an applicable malignant or non-malignant reference label. The retained study records do not document an additional clinical sampling criterion beyond inclusion in this analysis set; therefore, no additional retrospective inclusion or exclusion criteria are inferred.

Image preprocessing

The ColonCancerCT-2025 dataset provides CT imaging prepared for computational analysis [20]. No investigator-directed lesion segmentation, bounding-box annotation, manual region-of-interest selection, or radiomic feature extraction was performed before model training. No additional preprocessing parameters beyond those documented in the source dataset and retained study workflow were assumed or retrospectively reconstructed. Images were uploaded to the automated image-classification platform with their corresponding malignant or non-malignant labels.

Dataset partitioning

The 3,827 images were divided into training, validation, and test partitions using an approximately 80:10:10 distribution. A total of 3,062 images were used for model training, 383 images were used for validation during model development, and 382 images were retained for final testing.

The training set was used by the automated platform for model development, while the validation set provided intermediate evaluation during training. The test set was withheld from model training and used for final performance assessment.

The retained study workflow does not establish that partitioning was performed at the patient level. Therefore, images from the same patient may have been distributed across the training, validation, and test sets. Because adjacent CT slices from a single examination can share highly similar anatomic, acquisition, and disease-related features, such patient-level overlap could introduce information leakage between dataset partitions. This represents the principal methodological limitation of the study and may have substantially inflated the reported performance estimates by allowing the model to encounter highly correlated images from the same underlying patient during both development and evaluation. Accordingly, the reported test-set performance should be interpreted as image-level performance within this dataset rather than as a reliable estimate of performance in completely unseen patients. Future studies should use strict patient-level partitioning to ensure that all images from a given patient are confined to a single dataset partition.

Model development and software

Model development and evaluation were performed using Google Cloud Vertex AI AutoML Image Classification (Google LLC, Mountain View, CA, USA), accessed in 2026. Vertex AI is a cloud-managed machine-learning platform; therefore, a conventional locally installed software version was not applicable or recorded. The platform name, provider, year of use, model task, and available configuration settings are reported to identify the software environment used in this study.

A single-label image-classification configuration was selected so that each CT image was assigned to one of two mutually exclusive diagnostic categories: malignant or non-malignant. Feature learning, model optimization, and selection of underlying platform-controlled parameters were performed automatically by the AutoML system. The investigators did not manually construct the neural-network architecture or individually optimize conventional model hyperparameters. Detailed information regarding the final architecture and certain platform-controlled optimization parameters was not directly accessible to the investigators.

For each evaluated image, the trained classifier generated confidence scores corresponding to the malignant and non-malignant categories. The category associated with the higher prediction score represented the model-assigned classification. A confidence threshold of 0.50 was selected for the principal performance evaluation.

Performance metrics and statistical analysis

Model performance was evaluated using the 382-image test partition. Prespecified evaluation measures included overall average precision, precision, recall, class-specific average precision, precision-recall analysis, confidence-threshold analysis, and a normalized binary confusion matrix.

Average precision summarized classification performance across changing prediction thresholds. Precision represented the proportion of model-assigned positive classifications corresponding to the relevant reference category, whereas recall represented the proportion of reference-positive images correctly identified by the classifier.

A precision-recall curve was examined to characterize the relationship between precision and recall across prediction thresholds. A separate confidence-threshold analysis evaluated the behavior of precision and recall as the minimum required prediction confidence changed.

The normalized confusion matrix was used to characterize class-specific correct and incorrect classifications. When malignancy was considered the positive class, the normalized matrix demonstrated correct recognition of 97% of malignant images and correct exclusion of malignancy in 100% of non-malignant images, corresponding to a malignant-image sensitivity of 97% and non-malignant-image specificity of 100% within the normalized test output.

The retained Vertex AI evaluation output contained summary performance measures and normalized confusion-matrix percentages rather than the complete raw class-specific confusion-matrix counts and individual prediction-level data required for independent calculation of all additional statistics. Consequently, exact overall accuracy, ROC-AUC, and binomial 95% confidence intervals could not be validly reconstructed and were not estimated retrospectively. Average precision was not treated as interchangeable with ROC-AUC. No inferential hypothesis testing was performed; the analysis was descriptive and intended to characterize proof-of-concept image-classification performance.

Ethical considerations

This study involved secondary computational analysis of de-identified archival CT images obtained exclusively from the publicly available ColonCancerCT-2025 dataset [20]. No participants were prospectively recruited or contacted, no new patient information was collected, and the investigators did not access direct patient identifiers or protected health information.

Institutional review board approval was not obtained because the investigation was limited to secondary analysis of publicly available de-identified data and did not involve prospective human-subject participation. An informed-consent waiver was likewise not requested or granted because no new participant enrollment or identifiable patient-data collection occurred.

## Results

Overall classification performance

The final test set contained 382 abdominal CT images representing the malignant and non-malignant diagnostic categories. At the prespecified confidence threshold of 0.50, the classifier achieved an overall average precision of 0.998, with overall precision and recall of approximately 99%. These performance estimates reflect image-level classification within the present test set only and should not be interpreted as estimates of patient-level diagnostic performance, particularly because independence of patients across the training, validation, and test partitions could not be confirmed. These findings indicate high image-level classification performance within the evaluated test partition.

The precision-recall relationship remained near the upper portion of the performance space across most evaluated thresholds, with high precision maintained while recall remained close to its maximum across a broad range of operating points. Recall declined more substantially only when the required prediction confidence approached the highest values, as shown in Figure 1.

**Figure 1:**
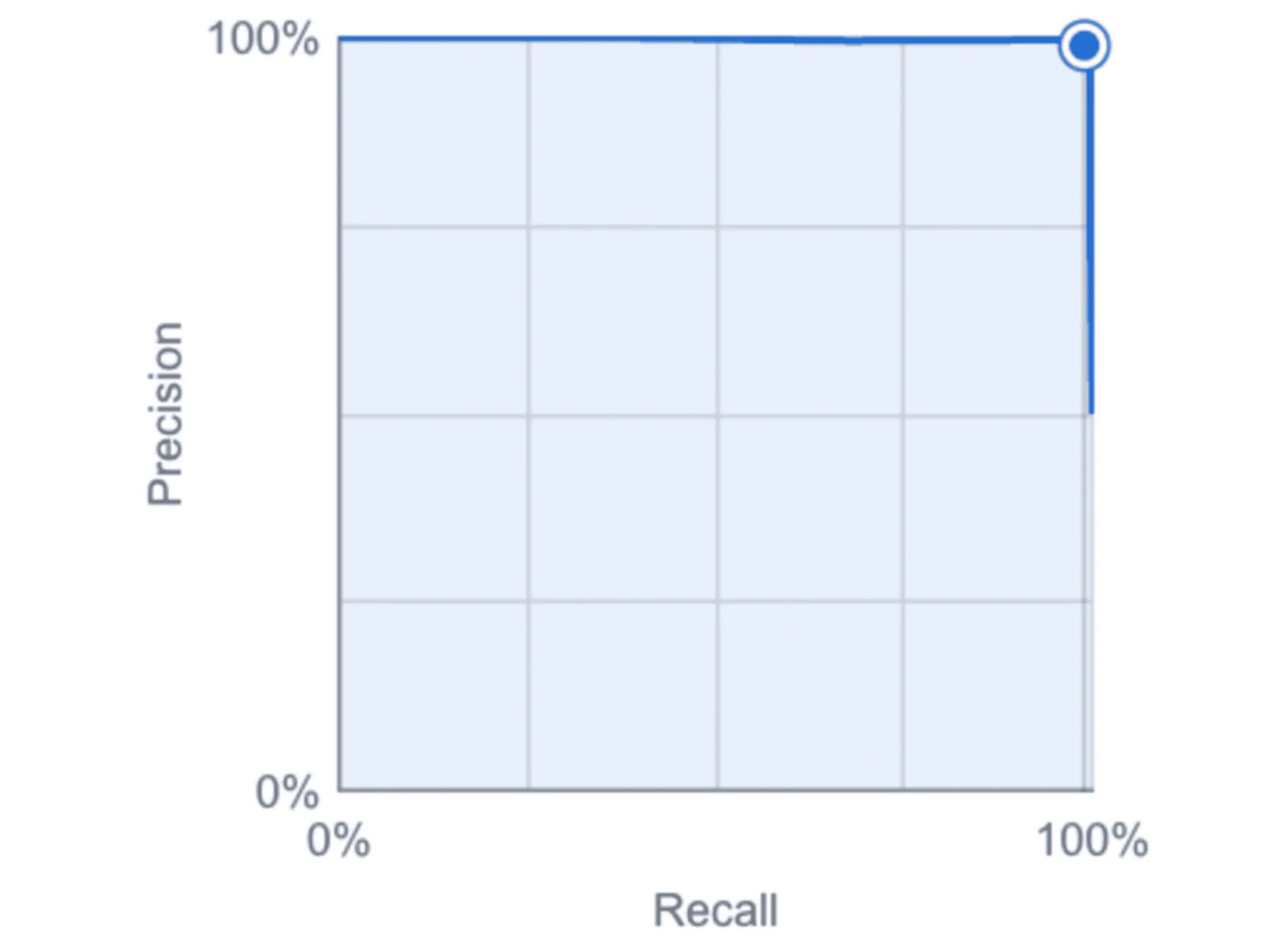
Precision-recall curve for malignant and non-malignant abdominal CT image classification. The curve demonstrates the relationship between precision and recall across prediction thresholds. The figure was generated from the Google Cloud Vertex AI AutoML model-evaluation output (Google LLC, Mountain View, CA, USA). Its location near the upper-right region of the plot reflects high image-level classification performance across most evaluated operating points. CT: computed tomography

Confidence-threshold analysis

The effect of changing the minimum prediction-confidence threshold was also evaluated. Precision increased rapidly at lower confidence thresholds and remained near its maximum across most of the evaluated range. Recall similarly remained high over a broad range before declining at highly restrictive confidence thresholds. At the selected confidence threshold of 0.50, precision and recall were both approximately 99%, as shown in Figure 2.

**Figure 2:**
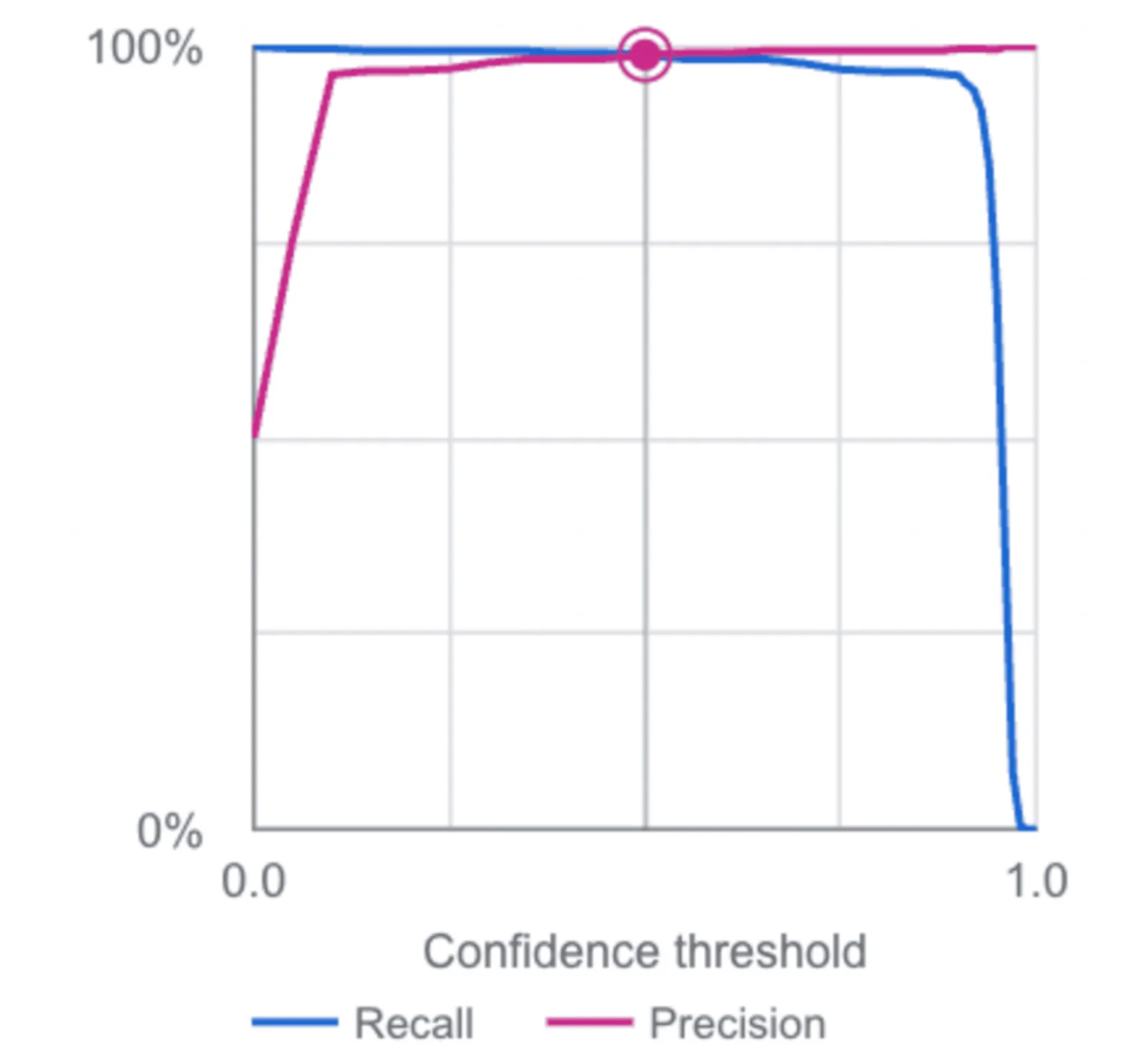
Precision and recall across confidence thresholds for the abdominal CT image-classification model. The figure was generated from the Google Cloud Vertex AI AutoML model-evaluation output (Google LLC, Mountain View, CA, USA). Precision and recall remained high across much of the evaluated confidence range, and both were approximately 99% at the prespecified confidence threshold of 0.50. CT: computed tomography

Class-specific performance and confusion matrix

Class-specific evaluation demonstrated similarly high average precision for both diagnostic categories. Average precision was 0.998 for malignant images and 0.999 for non-malignant images.

The normalized confusion matrix demonstrated that 97% of images carrying a malignant reference label were correctly classified as malignant, whereas approximately 3% were classified as non-malignant. All images carrying a non-malignant reference label were correctly classified as non-malignant in the normalized output. When malignancy was considered the positive category, these normalized results corresponded to a malignant-image sensitivity of 97% and a non-malignant-image specificity of 100%. The distribution of model classifications is shown in Figure 3.

**Figure 3:**
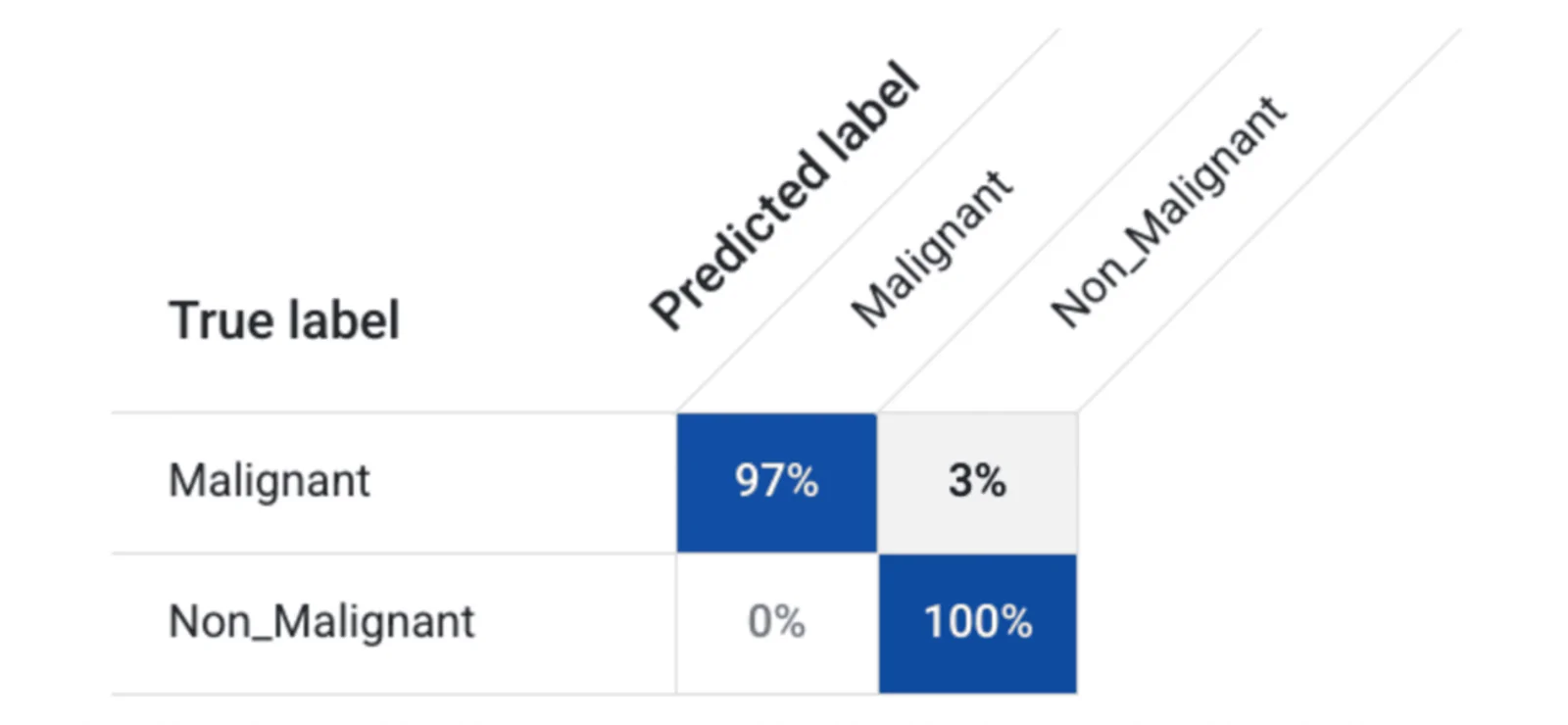
Confusion matrix for malignant and non-malignant abdominal CT image classification. The normalized confusion matrix was generated from the Google Cloud Vertex AI AutoML model-evaluation output (Google LLC, Mountain View, CA, USA) and compares reference image labels with model-predicted categories. The classifier correctly identified 97% of malignant images and 100% of non-malignant images. Approximately 3% of malignant images were classified as non-malignant. CT: computed tomography

The retained Vertex AI evaluation output provided summary performance measures and normalized confusion-matrix percentages rather than complete raw class-specific confusion-matrix counts and individual prediction-level data. Therefore, exact overall accuracy, ROC-AUC, and corresponding 95% confidence intervals could not be validly reconstructed and were not retrospectively estimated. Average precision was not treated as equivalent to ROC-AUC.

Representative image-level predictions

Representative correctly classified CT images were examined to demonstrate individual model predictions. For a representative non-malignant image, the classifier assigned a probability of 0.960 to the non-malignant category and 0.040 to the malignant category. For a representative malignant image, the model assigned a probability of 0.947 to the malignant category and 0.053 to the non-malignant category. In both examples, the reference category received the higher prediction probability, as shown in Figure 4.

**Figure 4:**
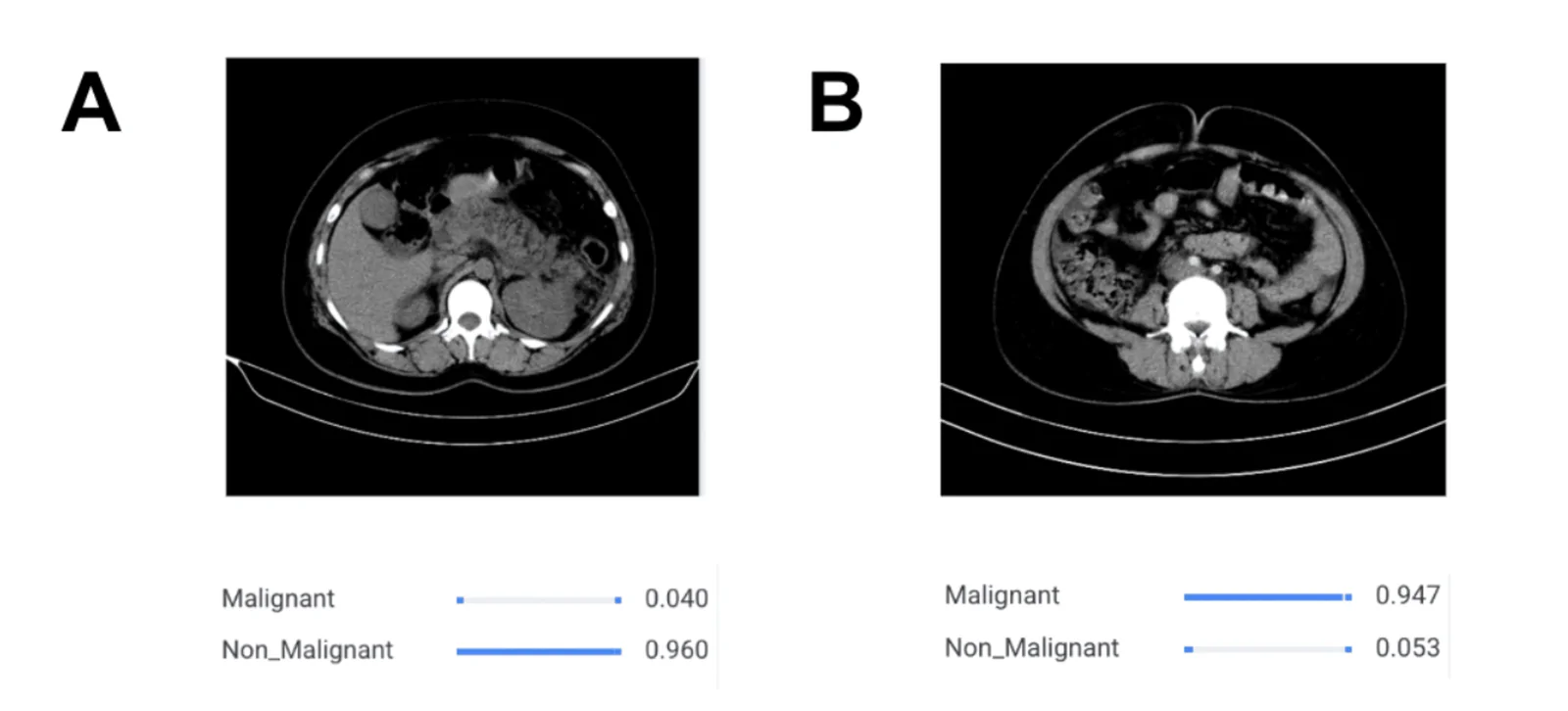
Representative artificial intelligence classifications of abdominal CT images. The composite was created using representative CT images from the ColonCancerCT-2025 dataset [20] and their corresponding Google Cloud Vertex AI AutoML prediction-confidence outputs (Google LLC, Mountain View, CA, USA). Panel (A) demonstrates a correctly classified non-malignant image, for which the classifier assigned a probability of 0.960 to the non-malignant category and 0.040 to the malignant category. Panel (B) demonstrates a correctly classified malignant image, for which the classifier assigned a probability of 0.947 to the malignant category and 0.053 to the non-malignant category. CT: computed tomography

## Discussion

This proof-of-concept study evaluated a cloud-based AutoML image-classification model for distinguishing malignant from non-malignant abdominal CT images. Within the evaluated dataset, the model demonstrated high image-level performance. At a confidence threshold of 0.50, overall average precision reached 0.998, with precision and recall of approximately 99%. Class-specific average precision was similarly high, measuring 0.998 for malignant images and 0.999 for non-malignant images. The normalized confusion matrix demonstrated correct classification of 97% of malignant images and 100% of non-malignant images. Taken together, these findings indicate that the model was able to distinguish the two image categories with few observed classification errors within the available test partition.

The distribution of classification errors provides additional information regarding model behavior. All non-malignant images were correctly categorized in the normalized confusion matrix, whereas approximately 3% of malignant images were assigned to the non-malignant class. Thus, the observed errors predominantly involved missed malignant image classifications rather than false-positive malignant classifications. Potential contributors include differences in lesion conspicuity, tumor size, tumor morphology, contrast characteristics, surrounding anatomy, reconstruction characteristics, or visual similarity between malignant and non-malignant findings. However, lesion-level annotations and explainability analyses were not available, preventing determination of which specific imaging characteristics contributed to individual errors.

The precision-recall and confidence-threshold analyses further demonstrated apparently stable model behavior within the evaluated test partition. However, formal probability calibration was not assessed because the retained model-evaluation output did not include the individual prediction-level data required for calibration analysis. Consequently, the reported confidence scores should not be interpreted as calibrated estimates of malignancy probability. Future validation studies should include formal calibration assessment, such as calibration plots and Brier scores, before clinical implementation of the model. Precision and recall remained high throughout much of the threshold range, with recall declining more substantially only when the minimum required prediction confidence approached the upper end of the scale. The selected threshold of 0.50 therefore provided a favorable balance between precision and recall within the available data. Representative predictions were consistent with these aggregate findings, with prediction probabilities of 0.947 for a malignant example and 0.960 for a non-malignant example. Nevertheless, high confidence scores for individual predictions should not be interpreted as evidence that model probabilities are clinically calibrated because formal calibration analysis was not performed.

The present findings are consistent with a growing literature demonstrating the ability of AI to identify colorectal malignancy on CT. Yao et al. developed a deep-learning system for CRC detection using contrast-enhanced CT examinations performed without bowel preparation and demonstrated strong discrimination across internal, external, and real-world cohorts [11]. Their reader experiments also demonstrated improved radiologist performance with AI assistance, supporting the potential role of AI as an adjunct rather than an autonomous replacement for radiologic interpretation [11]. Chen et al. subsequently evaluated a deep-learning approach for CRC detection using routine non-contrast CT across multicenter and international populations and similarly reported strong performance across independent cohorts and improved radiologist performance with AI assistance [12]. These investigations provide stronger assessments of generalizability because they incorporated independent or multicenter testing, whereas the present study was limited to image-level testing within a single publicly available dataset.

AI-based CT analysis has also been evaluated for clinical tasks extending beyond primary tumor recognition. Bedrikovetski et al. investigated deep learning for prediction of lymph-node status from preoperative staging CT in patients with colon cancer, demonstrating both the feasibility and challenges associated with prediction of more complex clinical outcomes [13]. Radiomics and machine-learning approaches have additionally been studied for postoperative prognosis [14], recurrence and disease-free survival [15], cancer-specific survival [16], and prediction of primary tumor and nodal stages [17]. Collectively, these studies suggest that quantitative CT analysis may contain clinically relevant information extending beyond visually apparent tumor detection.

The present study differs from many manually developed deep-learning systems through its use of the Google Cloud Vertex AI AutoML image-classification platform. Automated machine-learning platforms may reduce technical barriers associated with manually designing neural-network architectures, configuring computational environments, and implementing model-training pipelines. This accessibility may facilitate proof-of-concept medical-imaging research among investigators who do not routinely develop neural networks from source code. However, automation also reduces investigator access to certain architectural and optimization details. Because portions of the model architecture and optimization process are controlled internally by the managed platform, complete technical reproducibility may be more difficult than with a fully investigator-defined model. Transparent reporting of the platform, data source, task configuration, dataset partitioning, operating threshold, and available evaluation metrics is therefore particularly important.

The most important limitation of the present study is the relationship between the number of images and the number of independent patients. Although 3,827 individual CT images were incorporated into the model-development workflow, these images originated from a source dataset containing only 40 patients, including 12 malignant and 28 non-malignant cases [20]. Thousands of individual CT slices should therefore not be interpreted as thousands of independent clinical observations. Multiple slices derived from the same examination may contain highly correlated anatomy, acquisition characteristics, and disease features. In addition, the present study did not incorporate model-explainability techniques. Future work should include approaches such as Gradient-weighted Class Activation Mapping (Grad-CAM) or similar visualization methods to determine whether model predictions are driven by clinically relevant colorectal image regions rather than unrelated or confounding features.

Furthermore, patient-level separation among the training, validation, and test partitions could not be confirmed. If images originating from the same patient were represented in more than one partition, the classifier could have encountered highly similar anatomical and imaging characteristics during both model development and testing. Such overlap can create information leakage and may inflate apparent test performance. Consequently, the reported average precision of 0.998 and precision and recall of approximately 99% should be interpreted strictly as image-level performance within the present workflow rather than as estimates of diagnostic performance in previously unseen patients. Future investigations should perform dataset partitioning at the patient level before individual images are assigned to training, validation, and test cohorts.

A second limitation is the small and imbalanced source population. Only 12 malignant and 28 non-malignant patients were represented in the source dataset [20]. Although class-specific average precision was high for both categories, such a limited patient population cannot adequately represent the heterogeneity of colorectal malignancy encountered in clinical practice. Tumor location, histologic subtype, disease stage, lesion size, enhancement pattern, bowel distention, patient characteristics, scanner manufacturer, reconstruction technique, and institutional imaging protocols may all influence CT appearance. Increasing the number of independent patients is therefore more important for evaluating generalizability than simply increasing the number of image slices derived from the same examinations.

Third, all images were obtained from a single publicly available data source, and no independent external dataset was used for evaluation. External validation is particularly important in medical imaging because models may unintentionally learn scanner-specific, institution-specific, or preprocessing-related characteristics rather than disease-specific imaging features. Future evaluation should therefore include datasets obtained from multiple institutions, scanner manufacturers, acquisition protocols, contrast phases, reconstruction parameters, and geographically diverse patient populations.

Fourth, the present classifier evaluated individual CT images rather than complete CT examinations. Clinical assessment of suspected colorectal malignancy does not depend on an isolated axial image. Radiologists integrate information from multiple contiguous slices, multiplanar reconstructions, contrast-enhancement patterns, prior imaging, clinical history, colonoscopic findings, laboratory information, and ultimately histopathologic confirmation. The image-level classification task evaluated in this study is therefore substantially simpler than real-world patient-level diagnosis, and the reported results should not be interpreted as equivalent to the diagnostic sensitivity, specificity, or accuracy of a complete clinical CT examination.

Fifth, the present study did not include direct comparison with radiologists or evaluation of AI-assisted reader performance. Previous investigations have demonstrated that the clinical usefulness of an imaging model may be better assessed by determining whether it improves physician performance rather than considering standalone algorithmic performance alone [11,12]. Future investigations should therefore include blinded reader studies involving radiologists with varying levels of experience and compare unaided interpretation, standalone AI classification, and AI-assisted interpretation.

Sixth, the classifier generated diagnostic-category predictions but did not provide lesion localization. It therefore remains uncertain whether classifications were based primarily on colorectal lesions, surrounding anatomy, acquisition characteristics, preprocessing-related features, or other correlated patterns. Future work incorporating lesion localization, segmentation, bounding-box annotations, or carefully validated explainability techniques could help establish whether model predictions are driven by clinically relevant image regions.

Finally, the retained analysis output contained normalized confusion-matrix percentages and summary performance measures rather than complete raw test-set prediction data. Accordingly, exact overall accuracy, ROC-AUC, class-specific raw confusion-matrix counts, and corresponding 95% confidence intervals could not be independently reconstructed. These measures were deliberately not estimated from insufficient information. Future studies should retain individual prediction scores and reference labels to permit complete statistical evaluation, including exact sensitivity, specificity, positive predictive value, negative predictive value, accuracy, ROC-AUC, precision-recall metrics, calibration measures, and confidence intervals.

Potential clinical implications and future directions

If the present findings are reproduced in appropriately separated and independently validated patient cohorts, AI-based CT classification could potentially serve as an adjunct to radiologic interpretation rather than as a replacement for physician assessment. Potential applications include identifying examinations containing suspicious colorectal findings for additional review, providing a secondary assessment when lesions are subtle, and assisting with prioritization of examinations in high-volume clinical workflows. Previous reader studies have demonstrated that AI assistance may improve radiologist performance in CRC detection, providing a rationale for evaluating similar human-AI collaboration with future versions of the present approach [11,12].

Clinical translation would require evaluation at the complete-examination and patient levels rather than on isolated CT images. Future studies should determine whether AI assistance improves radiologist sensitivity, specificity, diagnostic confidence, and interpretation efficiency across institutions and among readers with different levels of experience. Prospective evaluation would ultimately be required to establish whether any improvement in imaging performance results in earlier cancer recognition, changes in clinical management, or meaningful patient outcomes. These applications remain future research directions and should not be considered established clinical uses of the present proof-of-concept model.

Future investigations should prioritize substantially larger, independently curated, multi-institutional datasets with confirmed patient-level separation between training, validation, and test cohorts. Independent external testing should be performed using institutions not represented during model development. Further research should also progress from isolated image classification toward complete examination-level analysis, lesion detection and localization, segmentation, multiclass classification of colorectal abnormalities, tumor-staging prediction, and integration of imaging features with clinical information.

## Conclusions

The Cloud-based Google Cloud Vertex AI AutoML image-classification model demonstrated high image-level discrimination between malignant and non-malignant abdominal CT images within the evaluated dataset, achieving an overall average precision of 0.998 and approximately 99% precision and recall at a confidence threshold of 0.50, while the normalized confusion matrix demonstrated correct classification of 97% of malignant images and 100% of non-malignant images. These findings demonstrate the technical feasibility of automated cloud-based classification of colorectal CT images; however, they should be interpreted cautiously because the source dataset contained only 40 patients, patient-level separation among the training, validation, and test partitions could not be confirmed, independent external validation was not performed, and the model evaluated individual images rather than complete clinical CT examinations. The present system should therefore be considered a proof-of-concept research model rather than a validated diagnostic tool. Before any clinical deployment, future validation should be performed at both the patient and complete-examination levels using independent, multi-institutional datasets with strict patient-level separation between development and evaluation cohorts. Additional studies should incorporate lesion localization, radiologist comparison, comprehensive statistical and calibration assessment, and prospective clinical evaluation to determine whether performance can be reproduced in previously unseen patients and translated into meaningful clinical benefit.
